# Engineering of global transcription factor FruR to redirect the carbon flow in *Escherichia coli* for enhancing l-phenylalanine biosynthesis

**DOI:** 10.1186/s12934-022-01954-7

**Published:** 2022-10-26

**Authors:** Minliang Chen, Hengyu Liang, Chao Han, Peng Zhou, Zhiwei Xing, Qianqian Chen, Yongyu Liu, Gou-an Xie, Rufei Xie

**Affiliations:** 1 Henan Joincare Biopharma Research Institute Co. Ltd, Jinyuan Street 8, Jiaozuo, 454000 People’s Republic of China; 2 Jiaozuo Joincare Biotechnology Co. Ltd, Jinyuan Street 8, Jiaozuo, 454000 People’s Republic of China; 3 Guangdong Provincial Key Laboratory of Research and Development and Application of Fermentation and Semi-Synthetic Drugs, Livzon New North River Pharmaceutical Co. Ltd, 1st Renmin Road, Qingyuan, 511500 People’s Republic of China

**Keywords:** FruR, l-phenylalanine, Transcriptome, Metabolome, CRISPR/Cas9

## Abstract

**Background:**

The catabolite repressor/activator protein (FruR) is a global regulatory protein known to control the expression of several genes concerned with carbon utilization and energy metabolism. This study aimed to illustrate effects of the FruR mutant on the _L-_phenylalanine (_L-_PHE) producing strain PHE01.

**Results:**

Random mutagenesis libraries of *fruR* generated in vitro were first integrated into the chromosome of PHE01 by CRISPR/Cas9 technique, and then the best mutant PHE07 (FruR^E173K^) was obtained. With this mutant, a final _L-_PHE concentration of 70.50 ± 1.02 g/L was achieved, which was 23.34% higher than that of PHE01. To better understand the mechanism, both transcriptomes and metabolomes of PHE07 were carried out and compared to that of PHE01. Specifically, the transcript levels of genes involved in gluconeogenesis pathway, pentose phosphate pathway, Krebs cycle, and glyoxylate shunt were up-regulated in the FruR^E173K^ mutant, whereas genes *aceEF, acnB*, and *icd* were down-regulated. From the metabolite level, the FruR^E173K^ mutation led to an accumulation of pentose phosphate pathway and Krebs cycle products, whereas the products of pyruvate metabolism pathway: acetyl-CoA and *cis*-aconic acid, were down-regulated. As a result of the altered metabolic flows, the utilization of carbon sources was improved and the supply of precursors (phosphoenolpyruvate and erythrose 4-phosphate) for _L-_PHE biosynthesis was increased, which together led to the enhanced production of _L-_PHE.

**Conclusion:**

A novel strategy for _L-_PHE overproduction by modification of the global transcription factor FruR in *E. coli* was reported. Especially, these findings expand the scope of pathways affected by the *fruR* regulon and illustrate its importance as a global regulator in _L-_PHE production.

**Supplementary Information:**

The online version contains supplementary material available at 10.1186/s12934-022-01954-7.

## Background

l-phenylalanine (_L-_PHE), which is an essential amino acid, has wide applications in food, agricultural, and pharmaceutical industries, and it is also an important chiral substrate for the synthesis of the low-calorie sweetener aspartame (l-phenylalanyl-l-aspartyl-methyl ester) [1, 2]. In recent years, as one of the aromatic amino acids, _L-_PHE production via metabolically engineered microbes, e.g., *Corynebacterium glutamicum* [3] and *Escherichia coli* [4, 5], has become more promising than other production routes, e.g., chemical synthesis or hydrolytic cleavage of proteins.

In *E. coli*, the biosynthetic pathway of _L-_PHE can be divided into two parts: the chorismate pathway and the _L-_PHE branch. The chorismate pathway connects the glycolysis and the pentose phosphate pathway and ends in the formation of chorismate. It begins with the condensation of the phosphoenolpyruvate (PEP) and erythrose 4-phosphate (E4P) to form 3-deoxy-d-arabinoheptulosonate-7-phosphate (DAHP). In the _L-_PHE branch, _L-_PHE is produced from chorismate in two steps catalyzed by the enzymes encoded by *pheA* and *tyrB* [6]. Till now, considerable attention has been paid to the sustainable production of _L-_PHE in microbial cells using the strategies of rational metabolic engineering, including (i) alleviation of all restrictive regulations [7–9]; (ii) deletion of competing pathways [5]; (iii) enhancement and balancing of precursor supplements [10, 11]; and (iv) removal of _L-_PHE degradation pathway. However, current strategies are mainly focused on the engineering of _L-_PHE biosynthetic pathway itself, and it is challenging to generate a high production of the desired chemical by these classical engineering approaches [5]. In recent years, transcriptional engineering approaches have been applied to strain optimization, which makes the engineering process carried out at a global and systematic level [12–14]. The global regulation of metabolic networks through multiple transcription factors (TFs) is one of the complex mechanisms for prokaryotes to respond to the intracellular perturbations by altering the expression of related genes [15, 16]. As an important component of gene expression regulation, modification of TFs can cause changes in carbon flux in the relevant metabolic pathways; thus, engineering of TFs has been proved to be useful for redirecting the fluxes toward the desired pathway for improving the target component [17–19]. However, to the best of our knowledge, few work has been reported to improve the _L-_PHE biosynthesis by TFs engineering.

FruR (catabolite repressor/activator, also known as Cra), is known to regulate the expression of several genes concerned with carbon utilization and energy metabolism [20–22]. FruR is a dual transcriptional regulator and it modulates the direction of carbon flow by transcriptional activation of genes encoding enzymes concerned with the Krebs cycle and the glyoxylate shunt and by repression of those genes that are involved in the glycolytic and Entner-Doudoroff (ED) pathways [23, 24]. In a previous study, the influence of *fruR* knockout on tryptophan biosynthesis was reported [17], and the metabolomics analysis showed that *fruR* knockout significantly enhanced the metabolic flow toward glycolysis, pentose phosphate pathway, and TCA cycle, increasing levels of critical precursors and substrates for _L-_tryptophan biosynthesis. A similar result was also reported by Zeng group [9]. However, to our surprise, it was found that inactivating the global regulator FruR in our _L-_PHE-producing strain PHE01 led to a significant decrease in _L-_PHE production. This result revealed that the regulation effects of FruR are more complex than expected and it is worth exploring the mechanism further.

In this study, the functionality of the global regulator FruR for _L-_PHE over production was first verified. Then, CRISPR/Cas9-facilitated engineering was combined with sensor-guided in vivo screening for engineering of protein FruR. In order to clarify the regulation mechanism of FruR, comparison of multi-omics data was performed between the best mutant and the wild-type strains.

## Results and discussion

### Knockout of ***fruR*** affects the biosynthesis of _L-_PHE

To explore the impact of FruR on the biosynthesis of _L-_PHE, the gene *fruR* in our previously constructed _L-_PHE-producing strain PHE01/pCas9 was disrupted, generating the strain PHE03 (Table 4, details in Additional files 3). The capacity of _L-_PHE production of PHE03 was compared to that of strain PHE01 by carrying out shake-flask fermentations (Table 1).

Table 1 shows that the cultivation of both strains PHE01 and PHE03 obtained almost the same amount of biomass. However, at the end of fermentation, PHE03 produced 5.75 ± 0.55 g/L of _L-_PHE, which is 18.10% lower than that of the strain PHE01 (7.02 ± 0.23 g/L). Meanwhile, the productivity (Vp) and specific production rate (q_PHE_) were also significantly decreased. These results suggested that the disruption of regulator FruR could result in the imbalance of central carbon metabolism, and indirectly block the metabolic flow toward the _L-_PHE biosynthetic pathway. Also, this result demonstrated that the regulation functionality of protein FruR is critical for _L-_PHE overproduction, and this was not consistent with the reported case for L-tryptophan [9, 17]. To find out whether modification of the regulation functionality of protein FruR could increase the production of _L-_PHE, a random mutagenesis strategy combined with the _L-_PHE biosensor was employed for the engineering and characterization of FruR mutants.

**Table 1:** _L-_PHE fermentation parameters of the strains PHE01 and PHE03

Strain	OD610	_L-_PHE (g/L)	Vp (g/L/h)	q_PHE_ (mg PHE/g DCW/h)
PHE01	39.05 ± 0.56	7.02 ± 0.23	0.16 ± 0.05	11.95 ± 1.03
PHE03	36.50 ± 0.05	5.75 ± 0.55	0.13 ± 0.02	10.46 ± 0.81

The engineered _L-_PHE-producing strains were cultivated in 500 mL shake flasks for 43 h

The data represents the mean ± SD from three independent experiments

*Vp* volumetric productivity, *q*_*PHE*_ specific production rate of _L-_PHE

### Engineering and characterization of FruR mutants

To obtain a FruR mutant with a higher production of _L-_PHE, the *fruR* variants were generated in vitro by using the error-prone PCR, and then the resulting gene variants were integrated into the chromosome of PHE01/pCas9 using the CRISPR/Cas9 technique. Finally, the FruR mutants were screened and characterized with the aid of _L-_PHE biosensor and HPLC detection.

**Fig. 1:**
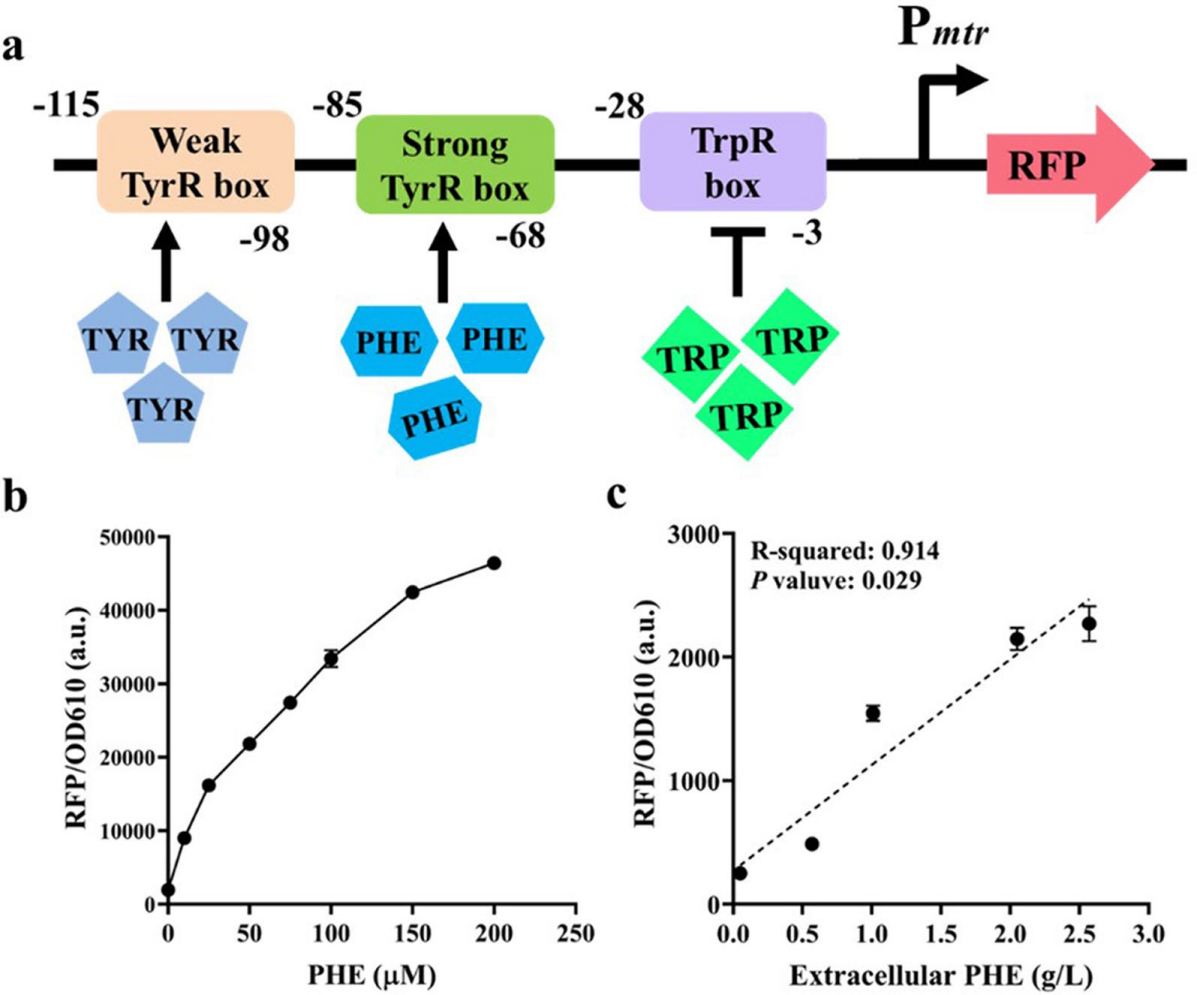
Library screening and characterization using an _L-_PHE biosensor. **a** Schematic illustration of the design of the _L-_PHE biosensor used in this study. The _L-_PHE biosensor (P*mtr*) is composed of a weak TyrR box (l-tyrosine binding site), a strong TyrR box (_L-_PHE binding site), and a TrpR box (l-tryptophan binding site). The biosensor regulates the expression of an engineered reporter (RFP) and placed upstream of the RFP reporter. **b** Fluorescence normalized by OD_610_ related to concentration of _L-_PHE supplemented into the media. **c** Extracellular _L-_PHE normalized by OD_610_ related to fluorescence normalized by OD_610_ (mean values with standard errors, *n* = 3 technical replicates). The *p*-value showing a significant slope is from a two-side *t*-test performed on means values for the five different strains, e.g., W3110, PHE01, PHE03, PHE04, and PHE05

Firstly, a host strain PHE01Δ*fruR*::*Cm*^*R*^ (Table 4, details in Additional files 3) was constructed by inserting a chloramphenicol resistance gene *Cm*^*R*^ into the locus of the *fruR* gene to offer the sgRNA target sequence for CRISPR/Cas9 application in further gene variant integration. In principle, an engineered FruR protein with a higher activity should lead to more accumulation of _L-_PHE, which in turn stimulate the expression of a report gene regulated by an _L-_PHE biosensor. The _L-_PHE biosensor is composed of the promoter of *mtr* gene (p*mtr*) [25] with RFP protein fused to the downstream (Fig. 1a). In detail, the plasmid backbone, upstream of the transcriptional start site of the p*mtr* promoter, and the DNA fragment encoding for RFP protein were amplified from the plasmid pBR332, the genomic DNA of *E. coli* K-12 W3110, and the template pET28a-RFP, respectively. All fragments were then pooled to an equimolar concentration and fused together to result in the final plasmid p*mtr*-RFP (details in Additional files 3). To characterize the designed biosensor, the plasmid p*mtr*-RFP expressed with the _L-_PHE biosensor was introduced into the host strain *E. coli* W3110. As observed in Fig. 1b, the _L-_PHE biosensor was able to activate RFP expression by a maximum of 23.86-fold upon supplementing _L-_PHE (0–200 μM) to the cultivation medium. To further validate the _L-_PHE biosensor, we measured fluorescence output in five different genotypes cultivated in the M9 medium, e.g., W3110, PHE01, PHE03, PHE04 (PHE01Δ*aroF*^*MT*^::*aroF*^*WT*^, details in Additional files 3), and PHE05 (PHE01Δ*pheA*^*MT*^::*pheA*^*WT*^, details in Additional files 3), and observed different biosensor outputs from these strains in line with the extracellular _L-_PHE concentration in strains with different genotypes (*R*^2^ = 0.914 and *p* = 0.029, Fig. 1c). These results demonstrated that the designed _L-_PHE biosensor has the ability to monitor changes in endogenously produced _L-_PHE pools.

Having established an _L-_PHE biosensor (Fig. 1), we next sought to apply the _L-_PHE biosensor for high-throughput screening and characterization of the FruR variants. To do so, PHE06 was constructed by introducing the plasmid p*mtr*-RFP into PHE01Δ*fruR*::*Cm*^*R*^/pCas9. Afterwards, a mutagenesis library of the *fruR* variant library was generated in vitro by using a Diversify^®^ PCR Random Mutagenesis Kit (PT3393-2, Takara Bio) and integrated into the chromosome of host PHE06 using the CRISPR/Cas9 technique. Finally, the mutants were screened and selected by using _L-_PHE biosensor-based in vivo characterization and HPLC-facilitated in vitro detection.

**Fig. 2:**
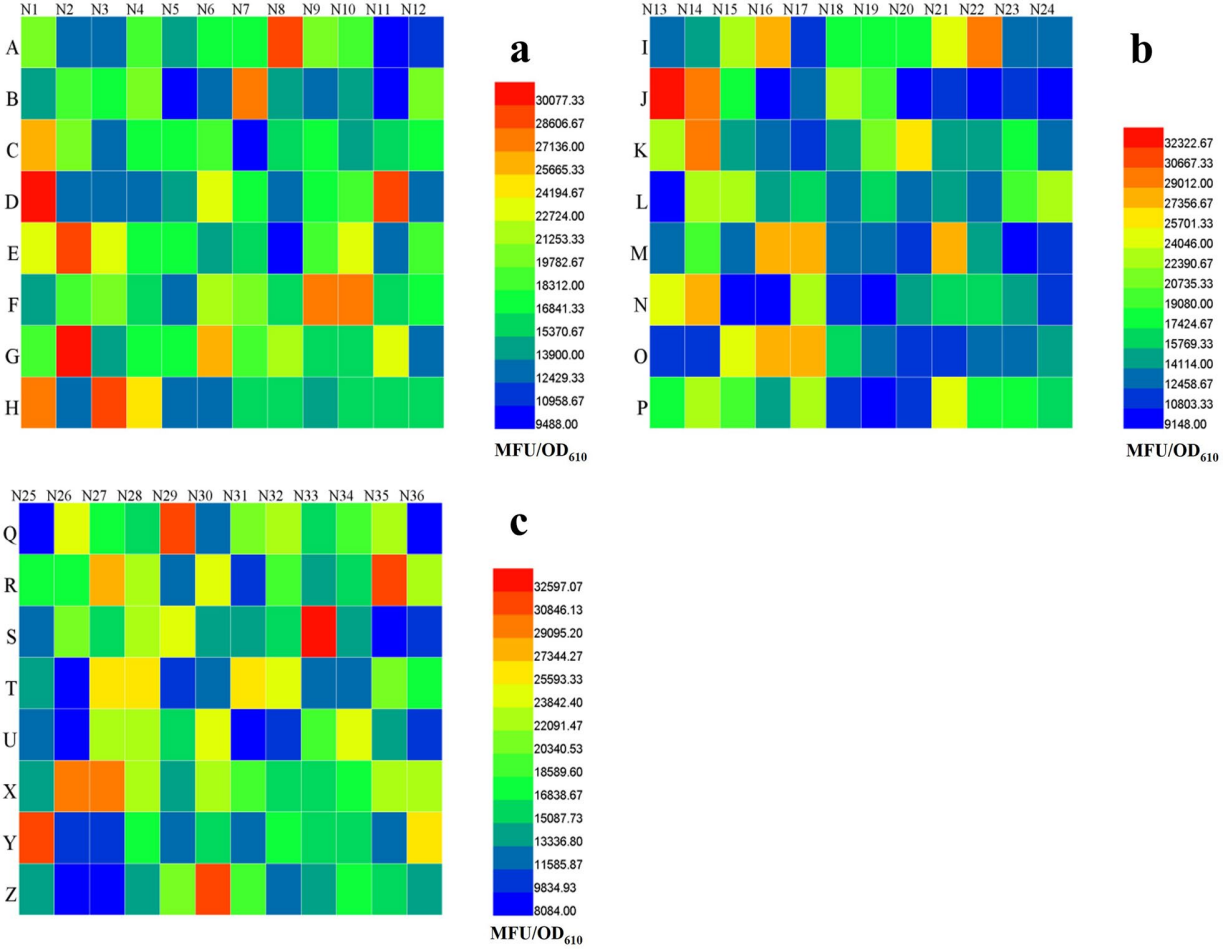
Heat maps of cell growth (OD_610_) and fluorescence intensity (MFU) of the selected mutants. A total of 276 samples in each well are presented as single colonies (**a**, **b**, and **c**). A total of 9 samples in H10-H12, P10-P12, and Z10-Z12 wells are presented as the controls: PHE01. The cells were cultured with fermentation medium in a 96-deep well plate

After the first-round screening, 279 colonies of the FruR mutants, which have relatively higher fluorescence signals, were selected and cultivated in three 96 deep-well plates. After 15 h of cultivation, cell growth and fluorescence intensity were measured and the results are presented in heat maps (Fig. 2). Among them, 50 mutants with a stronger fluorescence intensity (colored in red in Fig. 2) were isolated for sequencing. Sequencing results showed that there are 8 different types of FruR variants among these 50 candidates (Table 2), they are 40% for FruR^E173K^, 22% for FruR^A18P^, 16% for FruR^L3F–L170N^, 10% for FruR^S75I−V160E^, 6% for FruR^P129H^, 2% for FruR^I144T^, 2% for FruR^E72R−P128A^, and 2% for FruR^R312G^. Afterwards, all of these 8 types of recombinants were subjected to batch fermentation in shake flasks together with the positive control PHE01 (Table 2). As shown in Table 2, all of the selected FruR mutants had a higher _L-_PHE production compared to the strain PHE01. Especially, the FruR^E173K^ mutant exhibited the highest _L-_PHE production among all types of mutants. These results suggest that the variant FruR^E173K^ has a better performance for l-PHE production than the wildtype FruR^WT^ under the test conditions. Interestingly, the variant FruR^E173K^ was also generated by Zhang group in an _L-_PHE-producing strain HDH6-D12 by using random mutagenesis [26]. To provide more direct evidence, fed-batch fermentation was performed with the FruR^E173K^ mutant and the strain PHE01.

**Table 2:** Comparison of fermentation results with FruR mutants and PHE01

Mutants	Percentage ^a^	OD_610_	_L-_PHE (g/L)
FruR^E173K^	40	37.36 ± 0.25	9.06 ± 0.34
FruR^A18P^	22	36.50 ± 0.05	8.86 ± 0.62
FruR^L3F−L170N^	16	33.95 ± 1.05	7.79 ± 1.05
FruR^S75I−V160E^	10	34.43 ± 1.12	8.31 ± 0.67
FruR^P129H^	6	34.52 ± 2.15	8.46 ± 0.69
FruR^I144T^	2	35.12 ± 1.03	8.05 ± 0.42
FruR^E72R−P128A^	2	28.51 ± 1.35	7.69 ± 0.36
FruR^R312G^	2	27.35 ± 0.25	7.48 ± 0.47
PHE01^b^	–	36.06 ± 0.35	7.32 ± 0.35

^a^“Proportion” refers to the percentage of different types of mutants in all 50 candidates (%)

^b^Means the strain PHE01is used as a positive control; The fermentations were performed with a single clone for each FruR-variant

The average value ± standard deviation is based on three independent experiments

### Fed-batch fermentation of the variant FruR^E173K^

To explore the performance of the best variant FruR^E173K^ (PHE07) on enhancing the biosynthesis of _L-_PHE, the capacity of _L-_PHE production of the variant PHE07 was compared to that of the strain PHE01 by carrying out fed-batch fermentation in 50L bioreactors.

**Fig. 3:**
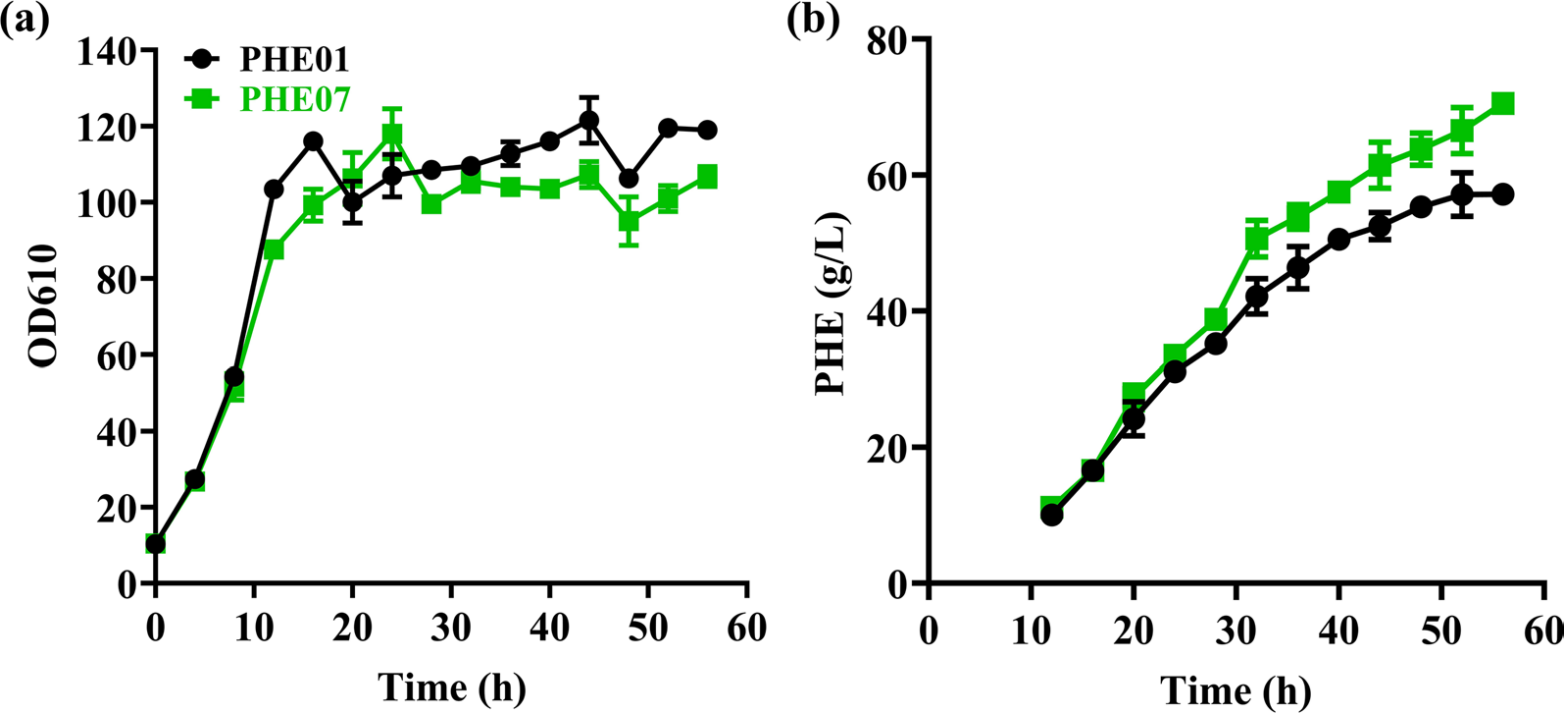
Fed-batch fermentation results of the strains PHE01 (black and circle) and PHE07 (green and square). **a** Cell growth; **b** PHE production. All results are based on two independent fermentations

As seen in Fig. 3a, the trend of cell growth of PHE07 was significantly lower than the strain PHE01. The overall cell biomass of the strains PHE01 and PHE07 were calculated to be 41.74 ± 0.49 g/L and 37.36 ± 0.17 g/L, respectively. However, as seen in Fig. 3b, the strain PHE07 produced a significantly higher amount of _L-_PHE than the reference strain PHE01 after the early stationary phase (about 20 h) until the end of the fermentation. At the end of fermentation (56 h), PHE07 produced 70.50 ± 1.02 g/L of _L-_PHE, which is 23.34% higher than that of the control PHE01 (57.16 ± 1.16 g/L, Table 3). According to Table 3, PHE01 and PHE07 consumed almost the same amount of glucose. It was thus suggested that in PHE07 a part of carbon flux was redirected from the central metabolic pathway to the biosynthesis of _L-_PHE. Considering the fact that FruR, as a global transcriptional regulator, mediates the flux balances among Krebs cycle, glyoxylate shunt, glycolytic pathway, gluconeogenesis pathway, and ED pathway [21], modification of the FruR protein could result in the flux being redirected toward the glyoxylate shunt and gluconeogenesis pathway, enhancing the supplying of precursors PEP and E4P for the biosynthesis of _L-_PHE.

**Table 3:** Comparison of the performances of the strains PHE01 and PHE07 for _L-_PHE production in fed-batch fermentation

Strains	OD_610_	GlcC^a^ (g/L)	_L-_PHE (g/L)	q_PHE_ (mg/g DCW/h)	Yield (g/g)	Vp (g/L/h)
PHE01	119.25 ± 1.42	342.27 ± 10.80	57.16 ± 1.16	24.45 ± 0.02	0.167 ± 0.001	1.02 ± 0.01
PHE07	106.75 ± 0.48	340.58 ± 11.20	70.50 ± 1.02	33.69 ± 0.13	0.207 ± 0.001	1.26 ± 0.01

Fed-batch fermentations were performed in 50 L bioreactors at 37 ℃ and pH 6.8; the initial glucose concentration was 10 g/L; the initial inoculation volume ratio was 0.25. Results are given as means ± standard deviations

^a^GlcC is the calculated cumulative consumption per reactor volume

Additionally, the overall specific formation rate of _L-_PHE in the strain PHE07 (33.69 ± 0.13 mg/g DCW/h, Table 3) showed an obvious advantage over that of the strain PHE01 (24.45 ± 0.02 mg/g DCW/h, Table 3). Also, it was notable that the yield of _L-_PHE for PHE07 was increased to 0.207 g/g at 56 h (Table 3), whereas it was 0.167 g/g for the reference strain PHE01. These results clearly demonstrated that expressing the FruR^E173K^ protein in the strain PHE07 was able to improve the specific production rate of _L-_PHE and the yield of _L-_PHE significantly (by approximately 37.79% and 23.95%, respectively). The performance of PHE07 in terms of the increased _L-_PHE production titer, rate, and yield makes it more attractive for industrial application. However, inconsistent with the previous reports [9, 17], overproduction of _L-_PHE was realized by expression of the FruR^E173K^ protein rather than knock-out of FruR in PHE07.

As seen in the schematic diagram of the interaction between FruR and its effector fructose-1,6-bisphosphate (FBP, Additional file 3: Fig. S6), the binding cavity is surrounded by residues Asn73, Tyr76, Arg197, Ser246, Phe247, Gln291, and Arg323. According to a previous study reported by Zhu et al., [14], modification of these binding sites resulted in decrease of the binding affinity between FruR and FBP [14]. In our case, E173K mutation might also result in a low protein-effector affinity, leading to enhancement of the binding affinity between FruR and its target genes. To this end, it was thus necessary to further explore the potential regulatory mechanism for FruR^E173K^ protein in the strain PHE07 by comparative transcriptomics and metabolomics analysis.

### Transcriptome and metabolome-wide effects of the regulator FruR in FruR^E173K^ in the _L-_PHE producer

**Fig. 4:**
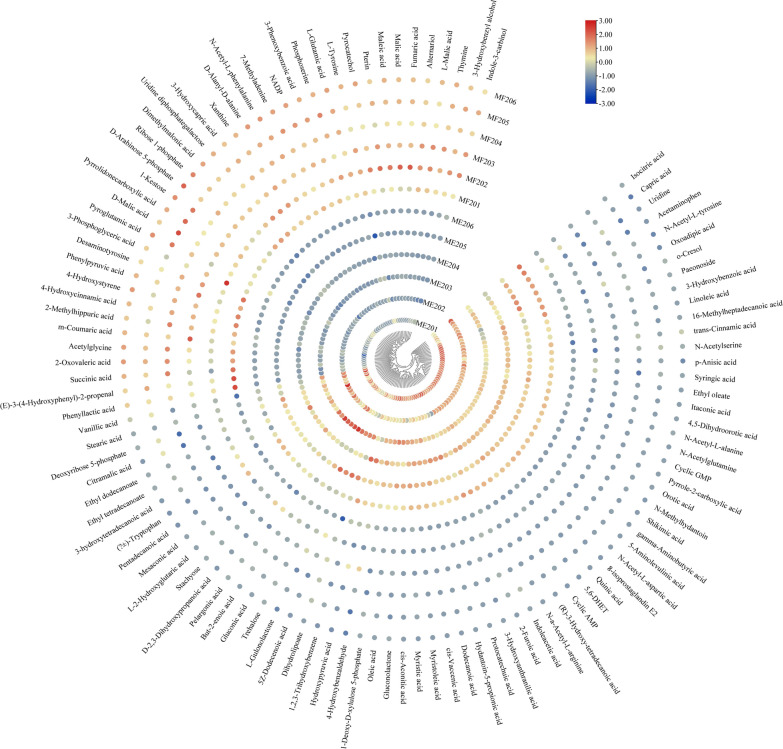
Heatmap of metabolite changes for the control group ME20 and the treatment group MF20 at 20 h of cultivation. Six independent biological repetitions of each group were measured and are shown in columns. Hierarchical clustering analyses were performed on those 12 samples with known annotations. The well in red indicates metabolite is up-expressed, and the well in blue indicates metabolite is down-expressed. Source data is available for this figure at Additional file 1: DataSet S1

To assess the influence of the FruR^E173K^ protein on gene expression and the composition of intracellular metabolites in the FruR^E173K^ mutant, transcriptomics and metabolomics analysis were carried out for the FruR^E173K^ mutant (MF) as well as the wildtype PHE01 (ME). For this purpose, both strains were cultivated in the modified _L-_PHE fermentation medium, and the samples were taken at the time point of 10 (Early-logarithmic phase), 20 (Mid-logarithmic phase), and 40 h (Stationary phase, Fig. 3a). In order to enable a direct comparison of the transcriptome and metabolome data, mRNA of the bacteria was isolated from the same culture samples as those used for intracellular metabolite extraction. According to the results of the metabolomics, 75 of the identified metabolites were significantly up-regulated and 98 of them significantly down-regulated (|fold change (fc)| ≤ 0.5 or  ≥ 1.5; corrected *p* ≤ 0.05) between the FruR^E173K^ mutant and the wildtype strain at the 20 h of cultivation (Fig. 4 and Additional file 1: Dataset S1). In parallel, transcriptomic data revealed that of 3143 profiled transcripts, 421 were significantly up-regulated and 224 significantly down-regulated in the FruR^E173K^ mutant compared to the wildtype strain, when a |log2fc| ≥ 2.0 and a corrected *p * ≤ 0.05 in gene expression were taken as cut-off (Fig. 6 and Additional file 2: DataSet S2).

**Fig. 5:**
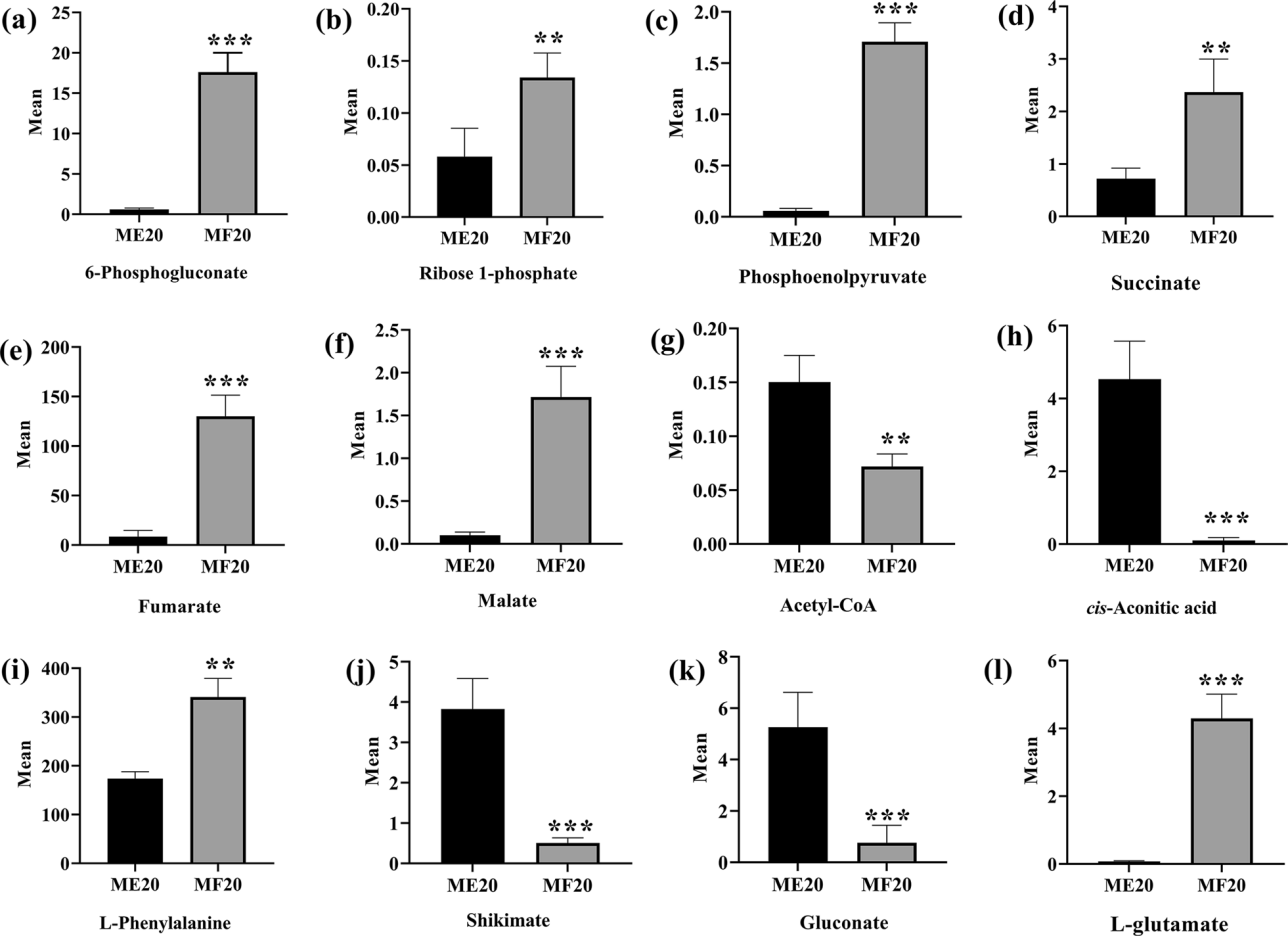
Levels of intermediates involved in L-phenylalanine biosynthesis were detected in the ME20 (PHE01) and MF20 (FruR^E173K^) at the cultivation of 20 h. *n* = 6 independent biological samples; **p < 0.01, ***p < 0.001

**Fig. 6:**
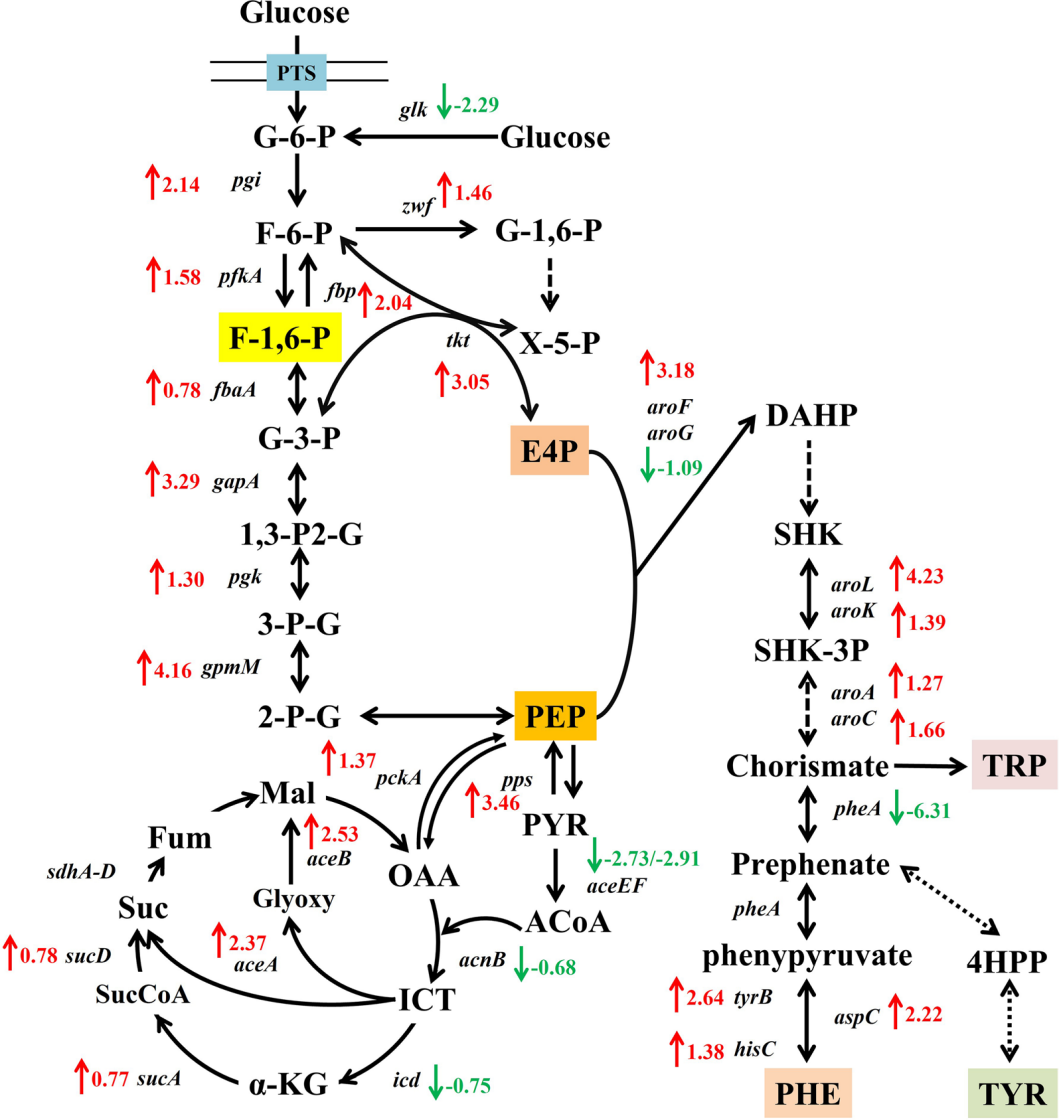
Regulations of central metabolic pathway and aromatic amino acid synthesis by the catabolites repressor/activator, Cra (FruR), in *E. coli* FruR^E173K^ mutant. The genes shown in aqua green boxes are under the control of FruR, some of which were identified by the experiments, and others were predicted by SELEX chip analyses or other prediction methods. The metabolite shown in a yellow box indicates the regulatory function of FruR is controlled by the intracellular concentration of the key metabolite, fructose-1,6-biphosphate. Green arrows indicate down-regulation, red arrows indicate up-regulation. Quantitative fold changes and corrected *p*-values are listed in Additional file 2: DataSet S2. Key genes for enzymes: Glucose-6-phosphate isomerase *pgi*, Fructose-1,6-bisphosphatase *fbp*, Phosphofructokinase *pfkA*, Fructose bisphosphate aldolase *fbaA*, Triose phosphate isomerase *tpi*, Glyceraldehyde 3-phosphate dehydrogenase-A *gapA*, Glyceraldehyde 3-phosphate dehydrogenase C *gapC*, Phosphoglycerate kinase *pgk*, Phosphoglycerate mutase M *gpmM*, Phosphoglycerate mutase A *gpmA*, Enolase *eno*, Pyruvate kinase I *pykF*, pyruvate kinase II *pykA*, Isocitrate lyase *aceA*, Malate synthase A*aceB*, Isocitrate dehydrogenase *icd*, 3-deoxy-d-arabino-heptulosonate-7-phosphate synthase *aroG/aroH*/*aroF*, 3-Dehydroquinate synthase *aroB*, 3-Dehydroquinate dehydratase *aroD*, Shikimate 5-dehydrogenase *aroE*, Shikimate dehydrogenase *ydiB*, Shikimate kinase II *aroL*, Shikimate kinase I *aroK*, 5-enolpyruvyl shikimate-3-phosphate synthase *aroA*, Chorismate synthase *aroC*, Bifunctional chorismate/prephenate dehydratase *pheA*/*tyrA*, Aromatic-amino-acid aminotransferase *tyrB*, Component of histidinol-phosphate aminotransferase *hisC*, Aspartate aminotransferase *aspC*

The most significant differences between the FruR^E173K^ mutant and the wildtype strain occurred in the central carbon metabolism (e.g., Krebs cycle, glyoxylate shunt, and glycolytic and gluconeogenesis pathway) and amino acid biosynthesis and metabolism (Figs. 4 and 5). The carbon flux balance between glycolytic and gluconeogenesis pathway is strictly regulated at a specific node in the EMP pathway by the regulator FruR: its effector fructose-1,6-bisphosphate (FBP) [27], meanwhile the metabolites in pentose phosphate pathway: 6-phosphogluconate (fc 261.07, *p* 8.88 × 10^–5^) and ribose 1-phosphate (fc 1.55, *p* 5.67 × 10^–4^), were up-regulated in the FruR^E173K^ mutant (Fig. 5a, b, Additional file 1: DataSet S1). On the transcriptome level (Fig. 6 and Additional file 2: DataSet S2), the enzymatic reactions of glycolytic and gluconeogenesis pathways were controlled correspondingly, especially the transcript levels of genes involved in the entire gluconeogenesis pathway (*gpmM*, *pfkA*, *gapA*, and *pgk*) were significantly up-regulated in the FruR^E173K^ mutant; In parallel, the transcript levels of genes associated with pentose phosphate pathway (*zwf* and *tkt*) were up-regulated (Fig. 6 and Additional file 2: DataSet S2). These results demonstrated that the modification of the regulator FruR probably activates the expression of genes involved in the gluconeogenesis pathway, which would convert the metabolic flux toward the pentose phosphate pathway and make the expression of genes associated with the pentose phosphate pathway were enhanced, and eventually contributing to the accumulation of E4P, a precursor to aromatic amino acid biosynthesis. Overexpression of the genes involved in the pentose phosphate pathway, such as *zwf* and *tkt*, has been widely applied for development of aromatic amino acids (AAAs)-producing strains [11, 28, 29], while overexpression of genes involved in the gluconeogenesis pathway (*gpmM*, *pfkA*, *gapA*, and *pgk*) for enhancing the accumulation of E4P is rare reported. Thus, these targets could be potential candidates for further improvements of _L-_PHE production, as well as other AAAs.

Corresponding metabolites could also be observed for the downstream Krebs cycle, where three metabolites (succinate, fumarate, and malate) were significantly up-regulated, while two metabolites (acetyl-CoA and *cis*-aconitic acid) were down-regulated in the FruR^E173K^ mutant (Fig. 5 and Additional file 1: DataSet S1). In line with this observation, parts of mRNAs of the involved enzymes from the Krebs cycle and glyoxylate shunt (*aceA*, *aceB*, *sucA*, and *sucD*) were activated (Fig. 6 and Additional file 2: Dataset S2), while the transcript level of genes (*aceEF*, *acnB*, and *icd*) was down-regulated in the FruR^E173K^ mutant, indicating that the transcriptome data supports our metabolome data. In addition, gene *pckA* (encoding phosphoenolpyruvate carboxykinase, |log2fc| 2.75, *p* 1.72 × 10^–21^) and gene *pps* (encoding phosphoenolpyruvate synthase, |log2fc| 3.40, *p* 3.45 × 10^–46^) were significantly up-regulated (Fig. 6 and Additional file 2: Dataset S2), which resulted in a strong accumulation of phosphoenolpyruvate (PEP, fc 19.15, *p* 0.03, Fig. 5 and Additional file 1: Dataset S1), a direct precursor to aromatic amino acid biosynthesis. These results revealed that FruR^E173K^ mutant activates the part of the Krebs cycle and the entire glyoxylate shunt; represses the metabolism of pyruvate and oxaloacetate and both of them are converted to PEP by enzyme Pps and PckA, respectively (Fig. 6), which resulted in the accumulation of PEP and eventually conducive to the biosynthesis of _L-_PHE. PEP, as a key node between the central metabolic pathway and the AAAs biosynthetic pathway, has been converted to AAAs biosynthesis by overexpression of the genes involved in the AAAs biosynthetic pathway (such as *aroG* [9], *aroF* [7], or *aroL* [30]), and the genes related to the central metabolic pathway (e.g., *pckA* and *pps* [11, 31]), or by knockout the gene *pykF* that encodes the pyruvate kinase I [11, 32]. Moreover, as revealed in transcriptome and metabolome data in this study, overexpression of genes involved in the glyoxylate shunt (*aceA* and *aceB*) and the succinate biosynthesis (*sucA* and *sucD*), or knockout of genes *aceEF*, *acnB*, and *icd*, might also be novel strategies for development of AAAs-producing strains.

In addition to the central carbon metabolism, the FruR^E173K^ mutant had a pronounced effect on the _L-_PHE biosynthesis pathway. As illustrated in Figs. 4 and 5, derived from the condensation of two molecules of PEP and E4P, a 94.66-fold decrease was found for shikimate (*p* 3.11 × 10^−5^), an intermediate of the central pathway for aromatic amino acids biosynthesis. This can be explained by the strong increase of the mRNA encoding the shikimate kinase AroL (|log2fc| 4.23, *p* 6.22 × 10^−133^) and AroK (|log2fc| 1.39, *p* 4.26 × 10^−11^), 5-enolpyruvyl shikimate-3-phosphate synthase *aroA* (|log2fc| 1.27, *p* 3.87 × 10^−9^), and chorismate synthase *aroC* (|log2fc| 1.66, *p* 4.19 × 10^−16^, Fig. 6 and Additional file 2: Dataset S2). Thus, it is possible that up-regulation of those genes in the FruR^E173K^ mutant led to a conversion of shikimate to the AAAs biosynthesis downstream, thereby increasing the biosynthesis of _L-_PHE (fc 19.15, Fig. 5). Hence, despite the central carbon metabolism, FruR^E173K^ mutant seems to also affect the AAAs pathway, especially activation of the downstream of the shikimate pathway and _L-_PHE pathway, which led to an accumulation of _L-_PHE.

As a member of the GalR-LacI family, FruR contains two functional domains: an N-terminal domain which contains a helix-turn-helix motif for DNA binding (AA 3-58 according to UniProt), and the effector binding domain located in the C terminus. FruR forms a complex with DNA in the absence of effector and acts as an activator of genes encoding gluconeogenic, Krebs cycle, and glyoxylate shunt enzymes. Upon the binding of the inducers, FruR is inactivated and the negative effect on genes encoding Entner-Doudoroff pathway and glycolytic enzymes is thus eliminated. In the previous reports on tryptophan bioproduction [17, 24], knockout of *fruR* results in function loss of the inducers and thus the genes involved in glycolysis, the Entner-Doudoroff pathway, and the PP pathway are activated and the genes involved in gluconeogenesis and the Krebs cycle are repressed. While in the case of _L-_PHE biosynthesis shown in this study, it was found that the changes in transcriptional levels of *fruR* gene between the FruR^E173K^ mutant (MF) and the wildtype PHE01 (ME) are not significant (Additional file 2: Dataset S2). Therefore, it is suspected that the effects of E173K mutation might be due to the decrease of the protein-effector binding affinity rather than the change of protein expression levels. As a consequence, the FruR^E173K^ mutant activates the gluconeogenesis pathway, alternative Krebs cycle, and the entire glyoxylate shunt, and no significant positive effect on the glycolysis and Entner-Doudoroff pathway was observed, as revealed by the metabolome and transcriptome analysis (Figs. 4 and 6). The different effects of FruR in the production of tryptophan and L-PHE might be due to the fact that more precursors are required for tryptophan biosynthesis. Besides the processors of PEP and E4P, PRPP and L-serine are also needed. Therefore, the balance among the pathways regulated by FruR should be modulated in different modes in order to achieve a high production. Another important reason may come from the different genetic backgrounds of the host strains, on which the functionality of the regulatory protein is highly dependent.

## Conclusion

Knockout of the *fruR* gene was first constructed in *E. coli* PHE01and the resulting strain PHE02 (PHE01Δ*fruR*) exhibited a reduced _L-_PHE production, indicating that the functionality of the global regulator FruR is necessary for _L-_PHE overproduction. To improve the _L-_PHE production of PHE01, CRISPR/Cas9-facilitated genome-integration of the *fruR* mutagenesis libraries was coupled with the biosensor-assisted library screening approach. The best mutant strain, PHE07 (FruR^E173K^), was obtained after several rounds of screening and characterization. Metabolomics and transcriptomics analysis suggested that the FruR^E173K^ mutant enhanced metabolic fluxes through the gluconeogenesis pathway, alternative Krebs cycle, the entire glyoxylate shunt, and the PP pathway, therefore channeling carbon fluxes to the _L-_PHE biosynthetic pathway. These altered metabolic flows not only improved the utilization of carbon sources, but also enhanced the supply of precursors (PEP and E4P) for _L-_PHE biosynthesis.

## Methods

### Strains and plasmids

The strains and plasmids used in this study are listed in Table 4. The primers used are summarized in Table S1. *E. coli* DH5α was used for plasmid construction. An _L-_tyrosine auxotrophic *E. coli* PHE01 was used as the host strain for expression and _L-_PHE production. To use the CRISPR/Cas9 technique for genome-editing or mutagenesis library genome-integration, the plasmid pCas9 [33] was introduced into PHE01, resulting in the strain PHE01/pCas9 (Table 4).

**Table 4:** Main strains and plasmids used in this study

Strains	Characteristics	Sources
*E. coli* W3110MT	W3110 derived mutant, l-tyrosine auxotrophic	Our lab
PHE01	W3110MT p15A::*pL*-*aroF*-*tyrA*^*1−121*^-*pR*-*pheA*^*T326P*^, kanamycin resistance	Our lab
PHE02	PHE01/pCas9, spectinomycin resistance	This work
PHE03	PHE01Δ*furR*	This work
PHE04	PHE01Δ*aroF*^*MT*^*:: aroF*^*WT*^	This work
PHE05	PHE01Δ*pheA*^*MT*^*:: pheA*^*WT*^	This work
PHE06	PHE01Δ*fruR::Cm*^*R*^*/*p*mtr-RFP*	This work
PHE07	PHE01Δ*furR::furR*^*E173K*^	This work
PHE08	PHE01Δ*furR::furR*^*A18P*^	This work
PHE09	PHE01Δ*furR::furR*^*L3F−L170N*^	This work
PHE10	PHE01Δ*furR::furR*^*S75I−V160E*^	This work
PHE11	PHE01Δ*furR::furR*^*P219H*^	This work
PHE12	PHE01Δ*furR::furR*^*I144T*^	This work
PHE13	PHE01Δ*furR::furR*^*E72R−P128A*^	This work
PHE14	PHE01Δ*furR::furR*^*R312G*^	This work
Plasmids		
pCas	expressing Cas9 protein and offering sgRNA for removing donor plasmid, Spectinomycin resistance	[33]
pGRB	plasmid for expressing sgRNA or with offering donor DNA, Ampicillin resistance	[33]
pGRB-Δ*fruR*	pGRB *fruR*-sgRNA^a^	This work
pGRB-*aroF*^*WT*^	pGRB *aroF*^*MT*^-sgRNA Δ*aroF*^*MT*^*::aroF*^*WT*^	This work
pGRB-*pheA*^*WT*^	pGRB *pheA*^*MT*^-sgRNA Δ*pheA*^*MT*^*::pheA*^*WT*^	This work
pGRB-Δ*fruR::Cm*^*R*^	pGRB *fruR*-sgRNA Δ*fruR::Cm*^*R*^	This work
pGRB-*fruR*^*MT*^	pGRB *Cm*^*R*^-sgRNA Δ*Cm*^*R*^*::fruR*^*MT*^	This work
p*mtr*-RFP	pBR332 p*mtr*-RFP (_L-_PHE biosensor)	This work

^a^*fruR*-sgRNA, sgRNA with an N20 sequence for targeting the *fruR* gene locus

### Implementation of CRISPR/Cas9 facilitated engineering and screening

#### Knocking-out of *furR* gene by using CRISPR/Cas9 technique

To realize the disruption of *fruR* gene, the strain PHE01/pCas9 (PHE02) was first constructed and used as the host strain. To achieve high efficiency for genome editing, the plasmid pGRB-Δ*fruR* (Additional file 3: Fig.S1) was constructed. This plasmid is able to offer the sgRNA targeting to the *fruR* gene and the homologous arms for recombination. More detailed information for the construction of pGRB-Δ*fruR* is presented in the Additional file 3.

We then followed the protocol reported by Chen and coworkers [34] to prepare the electroporation-competent cells and do the transformation. Specifically, an overnight culture (grown at 30 ℃) of the strain PHE02 was inoculated into 10 mL fresh SOC medium containing 50 μg/mL spectinomycin. After OD_600_ value reaching to 0.4–0.5, the cells were put on ice immediately for 10 min. Then, the cells were harvested by centrifugation at 4 ℃ and washed three times with precooled 10% glycerol. Competent cells were re-suspended in 400 μL precooled 10% glycerol and divided into 200 μL for each reaction. The corresponding sgRNA plasmid was mixed with the competent cells for transformation. The electroporation was done in the 0.2 cm cuvette at 2.5 kV, and the cells were suspended in 1 mL SOB medium and recovered for 2 h at 30 ℃ before plating. Plates were incubated more than 24 h at 30 ℃.

#### Construction of host strain for gene variant engineering and screening

To realize CRISPR/Cas9-facilitated library integration and in vivo screening, we first constructed a PHE02/p*mtr*-RFP (PHE03) strain in which an _L-_PHE biosensor was expressed in the plasmid p*mtr*-RFP (Additional file 3: Fig.S3). Afterward, the native FruR enzyme of the strain PHE02 was inactivated by replacing the *fruR* gene with chloramphenicol resistance gene (*Cm*^*R*^, as a selection maker), generating the strain PHE03Δ*fruR*::*Cm*^*R*^ (Table 4). The insertion of gene *Cm*^*R*^ expresses sgRNA sequence targets for further library integration using CRISPR/Cas9 technique.

#### Construction of gene variant library in vitro

To achieve genome-integration of mutagenesis library, it is necessary to construct a plasmid p*GRB*-*fruR*^*MT*^ (Additional file 3: Fig. S4), which contains the parts expressing the sgRNA targeting the *Cm*^*R*^ gene and the DNA fragment *fruR*^*MT*^ flanked by the corresponding homologous arms for recombination. Specifically, error-prone PCR of *fruR* gene was performed using a Diversify^®^ PCR Random Mutagenesis Kit (PT3393-2, Takara Bio) according to the manufacturer’s protocols. The purified PCR products were then ligated into the plasmid p*GRB*-*fruR*^*MT*^ using a seamless colony kit (D7010M, Beyotime). More detailed information is presented in the Additional file: 1, 2, 3.

#### Genome-integration and screening of *fruR* variant library

The *furR* variant library p*GRB*-*fruR*^*MT*^ was transferred into the competent cells of PHE03Δ*fruR*::*Cm*^*R*^ (containing pCas9). After incubation at 30 ℃ for more than 24 h, transformants with a stronger fluorescent signal were picked out and re-checked by streaking them on the same medium. Afterward, the candidate strains were tested by cultivation in 500 μL PHE-fermentation medium in 96-deep well plates at 30 ℃ for 24 h. Finally, the mutants giving higher medium fluorescent (MFU) and _L-_PHE concentration were selected for fermentation in shake flasks. Moreover, those mutants were selected for sequencing.

#### Method for measurement of fluorescent intensities

The mutants containing _L-_PHE biosensor with reporter RFP protein cultured in the LB medium were harvested by centrifugation and individually washed three times with the M9 medium to remove LB medium. Afterward, each mutant was inoculated with the same amount of cells into 10 mL fresh M9 medium in 50 mL conical tubes, and after cultivation of 10 h cells were subjected to fluorescence analysis using a multifunctional microplate reader. To this end, each culture was first washed three times using PBS buffer and diluted 100-fold and then RFP fluorescence was monitored using a microplate reader (Tecan M200 PRO) at an excitation wavelength of 540 nm. For fluorescent intensities, medium fluorescence unit (MFU) was calculated for each culture.

#### Fermentation

The fermentation medium with 1.5 g/L tyrosine was prepared as a previous report [28]. The condition for batch fermentation in shake flask was carried out in 500 mL conical flasks containing 50 mL fermentation medium inoculated with 5% (v/v) seed culture. All the batch fermentations were carried out at 37 ℃ and 250 rpm for 45 h. For fed-batch fermentation in the bioreactor, it was performed in a 50-L jar fermenter (BLBIO-10SJ-10SJ-50SJ-50SJ) with an initial broth volume of 20 L. The initial glucose concentration is 10 g/L, and it was maintained at 1–5 g/L by supplementing 700 g/L glucose in the fermentation process. Ammonia was used to maintain the pH at 6.8–7.0. The dissolved oxygen (DO) level was maintained at 25–30% saturation.

## Metabolite Analysis

### Sampling and LC–MS/MS analysis

Referring to the growth curve of PHE01 and PHE03 during the whole fermentation process (Fig. 3a), the samples in the time point of 10, 20, and 40 h were sampled and prepared for metabolites extraction (sextuplicate for each time point). Metabolite and transcript samples were taken simultaneously. The whole process took  ≤ 5 s (metabolites) or 10 s (transcripts) per sample from sampling to flash freezing in liquid nitrogen [35]. Metabolite extraction for liquid chromatography coupled to mass spectrometry (LC–MS) analysis was performed by resuspending the cell pellet with 500 µL ice-cold acetonitrile:methanol. The cell suspension was shock-frozen again in liquid nitrogen, and 500 µL of deionized water was added. Further metabolite extraction with repeating freeze–thaw-sonification cycles followed, as described previously [36].

LC–MS/MS analyses were performed using a UHPLC system (Vanquish, Thermo Fisher Scientific) with a UPLC BEH Amide column (2.1 mm × 100 mm, 1.7 μm) coupled to QExactive HFX mass spectrometer (Orbitrap MS, Thermo). The mobile phase consisted of 25 mmol/L ammonium acetate and 25 mmol/L ammonia hydroxide in water (pH = 9.75) (A) and acetonitrile (B). The QE HFX mass spectrometer was used for its ability to acquire MS/MS spectra on information-dependent acquisition mode in the control of the acquisition software (Xcalibur, Thermo). The ESI source conditions were set as following: sheath gas flow rate as 30 Arb, Aux gas flow rate as 25 Arb, capillary temperature 350 ℃, full MS resolution as 60,000, MS/MS resolution as 7500, collision energy as 10/30/60 in NCE mode, spray Voltage as 3.6 kV (positive) or −3.2 kV (negative), respectively [37].

### Data preprocessing and annotation

The raw data were converted to the mzXML format using ProteoWizard and processed with an in-house program, which was developed using R and based on XCMS, for peak detection, extraction, alignment, and integration [38, 39]. Then an in-house MS2 database (BiotreeDB) was applied in metabolite annotation. The cutoff for annotation was set at 0.3.

### Transcript analysis

#### RNA isolation, stand-specific library preparation, and Illumina sequencing

As mentioned previously, the samples in the time point of 10, 20, and 40 h were selected and prepared for RNA isolation (triplicate for each time point). Extraction of total RNA was carried out by the Invitrogen™ TRIzol™ Reagent Kit (15596018). RNA degradation and contamination were monitored on 1% agarose gels. Total amounts and integrity of RNA were assessed using the RNA Nano 6000 Assay Kit of the Bioanalyzer 2100 system (Agilent Technologies, CA, USA). Total RNA was used as input material for the RNA sample preparations. For our samples, mRNA was purified from total RNA by using probes to remove rRNA. Strand-specific RNA-seq cDNA library preparation of the total RNA of the different samples was based on RNA adapter ligation as described previously [40]. Afterward, the quality of the library was subsequently quantified by Qubit2.0 Fluorometer, Agilent 2100 bioanalyzer, and qRT-PCR. After the library is qualified, the different libraries are pooled according to the effective concentration and the target amount of data off the machine, then being sequenced by the Illumina NovaSeq 6000. The basic principle of sequencing is to synthesize and sequence at the same time (Sequencing by Synthesis). The fluorescent images measured by the high-throughput sequencer are converted into sequence data (reads) by CASAVA base recognition. Raw data (raw reads) of fastq format were firstly processed through in-house perl scripts [41].

#### Reading, mapping, bioinformatics, and statistics

Quality controlled and assessed libraries were mapped to the genome of *E. coli* strain W3110 (Acc.: chr: NC_007779) using Bowtie 2 (2.3.4.3) [42] with default parametrization. After read mapping, Rockhopper (1.2.1) was used to identify novel genes, operon, TSS, TTS, and Cis-natural antisense transcripts; RBSfinder (v1.0) [43] and TransTermH (2.0.9) [44] were used to predict SD sequence and terminator sequence, respectively; Rockhopper [45] and Blastx were used to annotate the newly predicted transgenic regions, and the unmarked transcripts were used as candidate non-coding sRNAs; RNAfold (1.8.5) [46] and IntaRNA (1.8.5) [47] were used to predict secondary structure and target gene, respectively.

The determined uniquely mapped read counts served as input to DESeq2 R package (1.20.0) [48] for pairwise detection and quantification of differential gene expression. The list of DESeq2 determined differentially expressed genes (DEGs) was filtered with a conservative cut-off: |log2fc| ≥ 1.5 and a corrected *p * ≤ 0.05. Gene Ontology (GO) enrichment analysis of differentially expressed genes was implemented by the ClusterProfiler R package (3.8.1) with pathway information from the KEGG database (http://www.genome.jp/kegg/). Results of the comparative transcriptome analysis are given in Additional file 2: DataSet S2.

## Supplementary Information

**Additional file 1:** DataSet 1-Differentially expressed metabolites between the FruRE173K mutant (MF) and the wildtype PHE01 (ME) at different periods of fermentation.

**Additional file 2:** DataSet 2-Comparisions of transcriptsome data between the FruRE173K mutant (MF) as well as the wildtype PHE01 (ME) at different periods of fermentation.

**Additional file 3: Figure S1.** The map of the plasmid pGRB-Δ*fruR*. This plasmid is composed of 1000 bp upstream and downstream of homologous arms for recombination and sgRNA-*fruR* sequence targeting to the *fruR* gene. **Figure S2.** The maps of the plasmids pGRB-*aroF*^*WT*^ (a) and pGRB-*pheA*^*WT*^ (b). **Figure S3.** The map of the PHE-biosensor p*mtr*-RFP. **Figure S4.** The map of the plasmid pGRB-*fruR*^*MT*^. This plasmid is composed of 1000 bp upstream and downstream of homologous arms for recombination, sgRNA-*CmR* sequence targeting to the *CmR* gene, and donor DNA fragment *fruR*^*MT*^. **Figure S5.** The map of the plasmid pGRB-Δ*fruR::Cm*^*R*^. This plasmid is composed of 1000 bp upstream and downstream of homologous arms for recombination, sgRNA-*fruR* sequence targeting to the *fruR* gene, and donor DNA fragment *CmR*. **Figure S6.** Details of the interaction between FruR and fructose-1,6-bisphosphate. Schematic diagram of protein-ligand interaction was generated by the LIGPLOT v.4.5.3. **Table S1.** Primers used in this study.

## Data Availability

Dataset that supports the findings of this study are available from the corresponding author upon reasonable request.
