# Supplementary material for: Engineering of global transcription factor FruR to redirect the carbon flow in Escherichia coli for enhancing l-phenylalanine biosynthesis
Source: Microb Cell Fact. 2022 Oct 26;21:222. doi: 10.1186/s12934-022-01954-7 (PMC9609185; doi:10.1186/s12934-022-01954-7)
Supplement: Supplementary file 3 — Additional file 3: Figure S1. The map of the plasmid pGRB-ΔfruR. This plasmid is composed of 1000 bp upstream and downstream of homologous arms for recombination and sgRNA-fruR sequence targeting to the fruR gene. Figure S2. The maps of the plasmids pGRB-aroFWT (a) and pGRB-pheAWT (b). Figure S3. The map of the PHE-biosensor pmtr-RFP. Figure S4. The map of the plasmid pGRB-fruRMT. This plasmid is composed of 1000 bp upstream and downstream of homologous arms for recombination, sgRNA-CmR sequence targeting to the CmR gene, and donor DNA fragment fruRMT. Figure S5. The map of the plasmid pGRB-ΔfruR::CmR. This plasmid is composed of 1000 bp upstream and downstream of homologous arms for recombination, sgRNA-fruR sequence targeting to the fruR gene, and donor DNA fragment CmR. Figure S6. Details of the interaction between FruR and fructose-1,6-bisphosphate. Schematic diagram of protein-ligand interaction was generated by the LIGPLOT v.4.5.3. Table S1. Primers used in this study. [file 12934_2022_1954_MOESM3_ESM.docx]

Supplementary Material

**Engineering the global transcription factor FruR to redirect the carbon flow in *Escherichia coli* for enhancing L-phenylalanine biosynthesis**

Minliang Chen^1,3*^, Hengyu Liang^1,2,3*,^ Chao Han^1,3^, Peng Zhou^1,3^, Zhiwei Xing^1,2^, Qianqian Chen^3^, Yongyu Liu^1^, Gou-an Xie^1^, Rufei Xie^1^

^1^ Henan Joincare Biopharma Research Institute Co. Ltd, Wanfang Industry Zone, Jiaozuo 454000, People’s Republic of China

^2^ Jiaozuo Joincare Biotechnology Co. Ltd, Wanfang Industry Zone, Jiaozuo 454000, People’s Republic of China

^3^ Guangdong Provincial Key Laboratory of Research and Development and Application of Fermentation and Semi-synthetic Drugs, Livzon New North River Pharmaceutical Co. Ltd, 1^st^ Renmin Road, Qingyuan 511500, People’s Republic of China

***Corresponding author**

E-mail address: [Minliang202208@163.com](mailto:Minliang202208@163.com) (Minliang Chen);

lhy-410@163.com (Hengyu Liang)

# Plasmids and strains construction

## **Construction of the donor DNA plasmid**

To construct the donor DNA plasmid expressing single guide RNA (sgRNA), a set of primers (Table Sl) was used to amplify the pGRB backbone (Fig. S1 and Table 4) through PCR. The 20bp spacer sequence specific for the target gene was selected using the web-based tool Cas-Designer (Bae et al., 2014; Park et al., 2015) and was synthesized within the primers (Table Sl, shown in capital letter). In detail, two 1000 bp homologous arms, DNA fragment for substitution, and the pGRB backbone were separately amplified and then fused together by a Seamless Cloning Kit (D7010M, Beyotime). After ligation, the PCR products were then transformed into *E. coli* Top10 competent cells directly to obtain the desired sgRNA plasmids.

## **Construction of the strain PHE03**

In order to knock out the gene *fruR* in the chromosome of the strain PHE02 by using the CRISPR/Cas9 technique, the plasmid pGRB-Δ*fruR* (Fig. S1) was first constructed. To this end, pGRB backbone was amplified by using the primer pairs *pGRB-REV*/*fruR-N20* (Table Sl) to obtain a *fruR*-sgRNA plasmid, pGRB-*fruRN20*. Afterward, the plasmid backbone, upstream and downstream homologous arms were amplified with the primer pairs sgRNA-REV/pGRB-FOR, *fruR*-out-FF/*fruR*-out-FR, and *fruR*-out-RF/*fruR*-out-RR (Table Sl), respectively. Then three fragments (V-N20*fruR*, F-*fruR*, and *fruR*-R) were fused together to construct the final plasmid pGRB-Δ*fruR* by using a Seamless Cloning Kit (D7010M, Beyotime). The plasmid pGRB-Δ*fruR* was transformed into the strain PHE02 with the plasmid pCas9 for generating the strain PHE03.


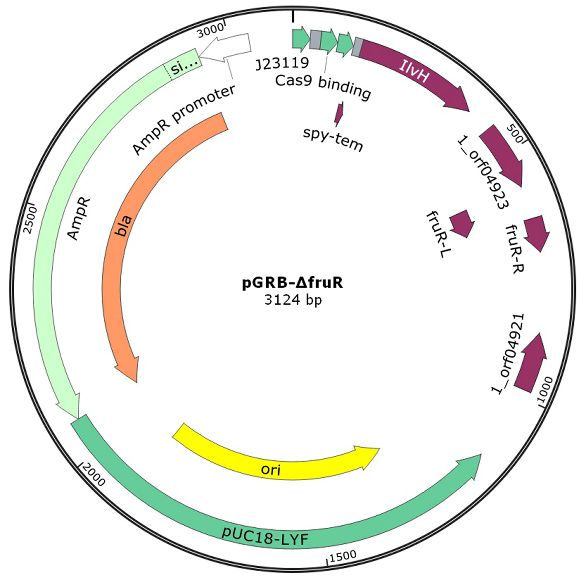


Figure S1. The map of the plasmid pGRB-Δ*fruR*. This plasmid is composed of 1000 bp upstream and downstream of homologous arms for recombination and sgRNA-*fruR* sequence targeting to the *fruR* gene.

## **Construction of the strains PHE04 and PHE05**

In order to construct different performances of PHE-producing strains, we replaced the feedback-resistant mutants PheA^fbr^ and AroF^fbr^ with the wildtype Phe^WT^ and AroF^WT^ in the chromosome of strain PHE01, respectively. In this regard, the plasmids pGRB-*aroFN20* and pGRB-*pheAN20* were constructed at first from the plasmid pGRB plasmid with the primer pairs *pGRB-REV*/*aroF-N20* and *pGRB-REV*/*pheA-N20* (Table S1), respectively, for expressing gRNA targeting the genes *aroF* and *pheA*. Then the plasmid backbone pGRB-*aroFN20*, the donor DNA fragment *F-aroF^WT^*, and the upstream and downstream homologous arms were amplified by using the primer pairs sgRNA-REV/pGRB-FOR, *aroF*-WT-FOR/ *aroF-WT-REV*, *aroF*-out-FF/*aroF*-out-FR, and *aroF*-out-RF/*aroF* -out-RR (Table S1), respectively. Afterward, four fragments (V-N20*aroF*, *aroF^WT^*, F-*aroF*, and *aroF*-R) were fused together to construct the final plasmid pGRB-*aroF^WT^* (Fig. S2a). Furthermore, the plasmid pGRB-*pheA^WT^* (Fig. S2b) was constructed in the same way as constructing the plasmid pGRB-*aroF^WT^* by using the corresponding primers (Table S1). The plasmid pGRB-*aroF^WT^* and pGRB-*pheA^WT^* were transformed into the strain PHE02 with the plasmid pCas9 for generating the strains PHE04 and PHE05, respectively.


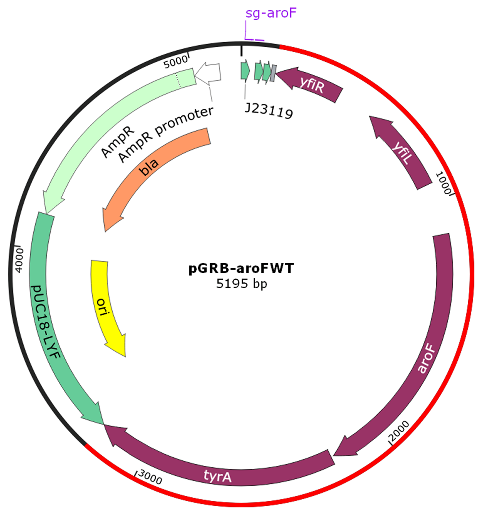

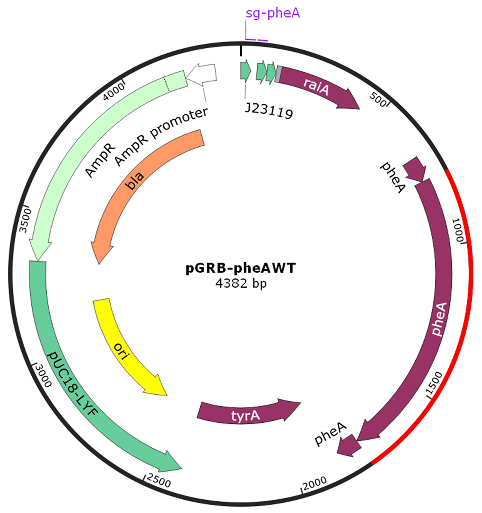


(b)）

(a)）

Figure S2. The maps of the plasmids pGRB-*aroF^WT^* (a) and pGRB-*pheA^WT^* (b).

## **Construction of PHE-biosensor** **p*mtr*-RFP**

For the construction of the PHE-responsive biosensor p*mtr*-RFP, the plasmid backbone pBR332 was amplified by using the primer pair *vector-FOR*/*vector-REV*, 331 bp upstream of the transcriptional start site of the P*mtr* promoter was amplified from the genomic DNA of *E. coli* K-12 W3110 using primer pair *pmtr-FOR*/*pmtr-REV* (Table Sl), and the DNA fragment encoding for RFP protein was amplified from pET28a-RFP with primer pair *RFP-FOR*/ *RFP-REV* (Table Sl). All fragments were pooled to an equimolar concentration and fused together to result in the final plasmid p*mtr*-RFP (Fig. S3).


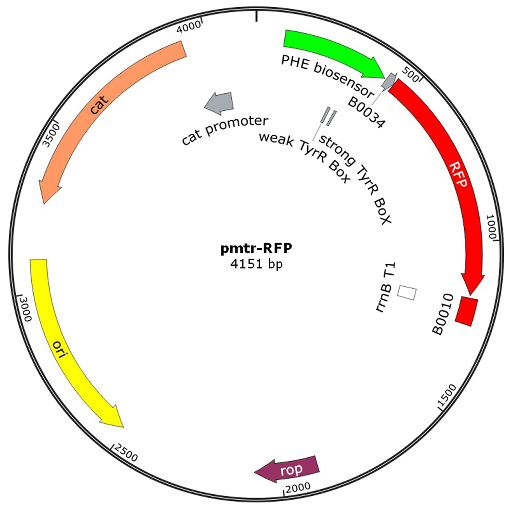


Figure S3. The map of the PHE-biosensor p*mtr*-RFP.

## **Construction of the plasmid libraries** **pGRB-*fruR^MT^***

To construct pGRB-*fruR^MT^* (Fig. S4), we constructed the plasmid p*Cm*N20-Δ*fruR* at first with the primers *pGRB-REV* and *Cm-N20* (Table S1) by using the plasmid pGRB-Δ*fruR* as template. Then the DNA fragment F-*fruR^MT^* was generated with the primer pairs *fruR-MT-FOR/fruR-MT-REV* (Table S1) and error-prone PCR of *fruR* gene was performed using a Diversify^®^ PCR Random Mutagenesis Kit (PT3393-2, Takara Bio) according to the manufacturer’s protocols; the plasmid backbone p*Cm*N20-Δ*fruR* was amplified with the primer pairs *fruR*-R-For-New/*fruR*-F-REV-New (Table S1). The purified PCR fragment F-*fruR^MT^* was then ligated into the plasmid backbone p*Cm*N20-Δ*fruR* to construct the plasmid libraries pGRB-*fruR^MT^* (Fig. S4).


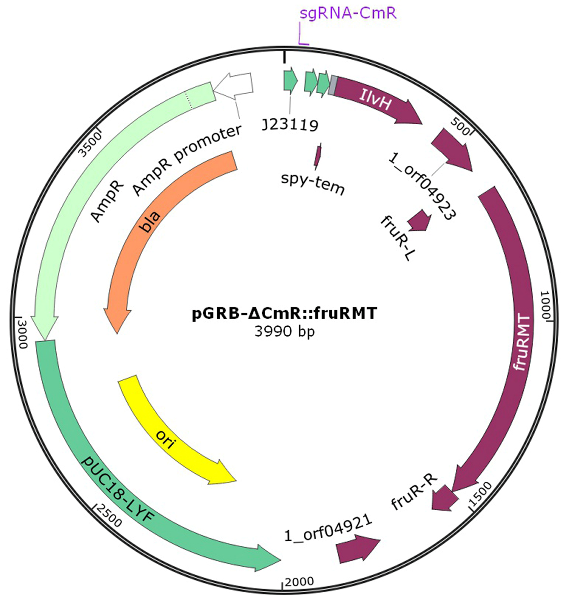


Figure S4. The map of the plasmid pGRB-*fruR^MT^*. This plasmid is composed of 1000 bp upstream and downstream of homologous arms for recombination, sgRNA-*CmR* sequence targeting to the *CmR* gene, and donor DNA fragment *fruR^MT^*.

## **Construction of host strain for** **gene variant engineering and screening**

To realize CRISPR/Cas9-facilitated *fruR* gene variants genome-integration and *in vivo* screening, the native FruR enzyme of the strain PHE02 was knocked out by replacing the fruR gene with chloramphenicol resistance gene (*Cm^R^*, as a selection maker), generating the strain PHE03Δ*fruR*::*Cm^R^* (Table 4). To do so, we constructed the plasmid pGRB-Δ*fruR::Cm^R^* (Fig. S5) at first by using the plasmid pGRB-Δ*fruR* as template. The plasmid backbone pGRB-Δ*fruR* was amplified with primer pair *fruR*-out-FR/*fruR*-out-RF (Table Sl) and the *Cm^R^* gene fragment was amplified from the plasmid pJLC with the primer pair *CmR-FOR* and *CmR-REV* (Table Sl). Then *Cm^R^* gene fragment was ligated into the plasmid pGRB-Δ*fruR* by using a Seamless Colony Kit to result in the plasmid pGRB-Δ*fruR::Cm^R^* (Fig. S5). Afterward, the plasmid pGRB-Δ*fruR::Cm^R^* was transformed into the strain PHE02 with the plasmid pCas9 for generating the strain PHE01Δ*fruR::Cm^R^*. Meanwhile, the PHE biosensor p*mtr*-RFP was transferred into the strain PHE01Δ*fruR::Cm^R^* to develop a host strain PHE06 (Table 4), which used for gene variant engineering and screening. Final, using the strain PHE06 as host strain, the gene variant library pGRB-*fruR^MT^* was integrated into the chromosome of the strain PHE06 with the aid of CRISPR/Cas9 technique for generating mutants PHE01Δ*furR::furR^MT^*.


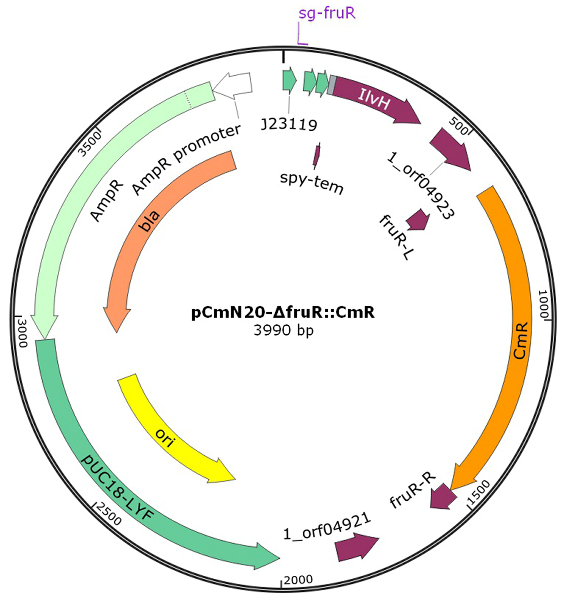


Figure S5. The map of the plasmid pGRB-Δ*fruR::Cm^R^*. This plasmid is composed of 1000 bp upstream and downstream of homologous arms for recombination, sgRNA-*fruR* sequence targeting to the *fruR* gene, and donor DNA fragment *CmR*.

## **Structure analysis and docking study**

To date, the available crystal structure for the FruR protein in complex with fructose 1-phosphate is only from *Pseudomonas putida* (PDB: 3O75). Therefore, a homology model was carried out using Modeller v9.25 with the crystal structure of *Pseudomonas putida* (PDB: 3O75) as the structural template [1]. Structural alignment was carried out with PyMOL. Docking of flexible ligand fructose-1,6-bisphosphate into the FruR protein was performed with AutoDock Vina [2]. Details of the interaction between FruR and fructose-1,6-bisphosphate is presented in Fig. S6.


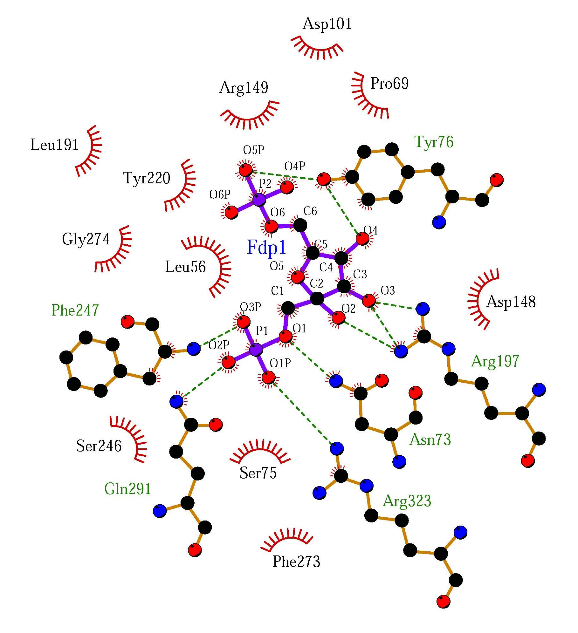


**Figure S6.** Details of the interaction between FruR and fructose-1,6-bisphosphate. Schematic diagram of protein-ligand interaction was generated by the LIGPLOT v.4.5.3.

## **p*mtr*-RFP Plasmid sequence**

TTCTCATGTTTGACAGCTTATCATCGATAAGCTTTAATGCGGTAGTTTATCACAGTTAAATTGCTAACGCAGTCAGGCACCGTGTgcagttactgggcgatgcacagccgcatactggcggtgagcgtcgtggcggtggtcgtggtttcggtggcgaacgtcgtgaaggcggtcgtaacttcagcggtgaacgccgtgaaggtggccgtggtgatggtcgtcgttttagcggcgaacgtcgtgaaggccgcgctccgcgtcgtgatgattctaccggtcgtcgtcgtttcggtggtgatgcgtaatcatcgctgaacagcgaacacaatctgtaaaataatatatacagccccgatttttaccatcggggctttttttctgtcttttgtactcgtgtactggtacagtgcaatgcataCTAGATACTAGAGAAAGAGGAGAAATACTAGATGGTTAGCAAAGGCGAAGAGCTGATCAAGGAGAACATGCACATGAAACTGTACATGGAAGGTACCGTTAACAACCACCATTTTAAGTGCACCAGCGAAGGCGAGGGTAAACCGTATGAGGGCACCCAGACCATGCGTATCAAGGTGGTTGAAGGTGGCCCGCTGCCGTTTGCGTTCGATATTCTGGCGACCAGCTTTATGTACGGCAGCCGTACCTTCATCAACCATACCCAGGGTATTCCGGATTTCTTTAAACAGAGCTTTCCGGAGGGCTTCACCTGGGAACGTGTGACCACCTACGAGGACGGTGGCGTTCTGACCGCGACCCAGGATACCAGCCTGCAAGACGGTTGCCTGATCTATAACGTGAAAATTCGTGGCGTTAACTTTCCGAGCAACGGTCCGGTGATGCAGAAAAAGACCCTGGGCTGGGAAGCGAACACCGAGATGCTGTACCCGGCGGATGGTGGCCTGGAAGGTCGTAGCGACATGGCGCTGAAGCTGGTTGGTGGCGGTCACCTGATCTGCAACTTCAAAACCACCTATCGTAGCAAAAAGCCGGCGAAAAACCTGAAGATGCCGGGCGTGTACTATGTTGATCATCGTCTGGAACGTATTAAAGAGGCGGACAAGGAAACCTACGTGGAACAGCACGAGGTGGCGGTTGCGCGTTATTGCGACCTGCCGAGCAAACTGGGTCATAAGCTGAACTAATACTAGTACCAGGCATCAAATAAAACGAAAGGCTCAGTCGAAAGACTGGGCCTTTCGTTTTATCTGTTGTTTGTCGGTGAACGCTCTCATGGAAGCCGGCGGCACCTCGCTAACGGATTCACCACTCCAAGAATTGGAGCCAATCAATTCTTGCGGAGAACTGTGAATGCGCAAACCAACCCTTGGCAGAACATATCCATCGCGTCCGCCATCTCCAGCAGCCGCACGCGGCGCATCTCGGGCAGCGTTGGGTCCTGGCCACGGGTGCGCATGATCGTGCTCCTGTCGTTGAGGACCCGGCTAGGCTGGCGGGGTTGCCTTACTGGTTAGCAGAATGAATCACCGATACGCGAGCGAACGTGAAGCGACTGCTGCTGCAAAACGTCTGCGACCTGAGCAACAACATGAATGGTCTTCGGTTTCCGTGTTTCGTAAAGTCTGGAAACGCGGAAGTCAGCGCCCTGCACCATTATGTTCCGGATCTGCATCGCAGGATGCTGCTGGCTACCCTGTGGAACACCTACATCTGTATTAACGAAGCGCTGGCATTGACCCTGAGTGATTTTTCTCTGGTCCCGCCGCATCCATACCGCCAGTTGTTTACCCTCACAACGTTCCAGTAACCGGGCATGTTCATCATCAGTAACCCGTATCGTGAGCATCCTCTCTCGTTTCATCGGTATCATTACCCCCATGAACAGAAATCCCCCTTACACGGAGGCATCAGTGACCAAACAGGAAAAAACCGCCCTTAACATGGCCCGCTTTATCAGAAGCCAGACATTAACGCTTCTGGAGAAACTCAACGAGCTGGACGCGGATGAACAGGCAGACATCTGTGAATCGCTTCACGACCACGCTGATGAGCTTTACCGCAGCTGCCTCGCGCGTTTCGGTGATGACGGTGAAAACCTCTGACACATGCAGCTCCCGGAGACGGTCACAGCTTGTCTGTAAGCGGATGCCGGGAGCAGACAAGCCCGTCAGGGCGCGTCAGCGGGTGTTGGCGGGTGTCGGGGCGCAGCCATGACCCAGTCACGTAGCGATAGCGGAGTGTATACTGGCTTAACTATGCGGCATCAGAGCAGATTGTACTGAGAGTGCACCATATGCGGTGTGAAATACCGCACAGATGCGTAAGGAGAAAATACCGCATCAGGCGCTCTTCCGCTTCCTCGCTCACTGACTCGCTGCGCTCGGTCGTTCGGCTGCGGCGAGCGGTATCAGCTCACTCAAAGGCGGTAATACGGTTATCCACAGAATCAGGGGATAACGCAGGAAAGAACATGTGAGCAAAAGGCCAGCAAAAGGCCAGGAACCGTAAAAAGGCCGCGTTGCTGGCGTTTTTCCATAGGCTCCGCCCCCCTGACGAGCATCACAAAAATCGACGCTCAAGTCAGAGGTGGCGAAACCCGACAGGACTATAAAGATACCAGGCGTTTCCCCCTGGAAGCTCCCTCGTGCGCTCTCCTGTTCCGACCCTGCCGCTTACCGGATACCTGTCCGCCTTTCTCCCTTCGGGAAGCGTGGCGCTTTCTCATAGCTCACGCTGTAGGTATCTCAGTTCGGTGTAGGTCGTTCGCTCCAAGCTGGGCTGTGTGCACGAACCCCCCGTTCAGCCCGACCGCTGCGCCTTATCCGGTAACTATCGTCTTGAGTCCAACCCGGTAAGACACGACTTATCGCCACTGGCAGCAGCCACTGGTAACAGGATTAGCAGAGCGAGGTATGTAGGCGGTGCTACAGAGTTCTTGAAGTGGTGGCCTAACTACGGCTACACTAGAAGGACAGTATTTGGTATCTGCGCTCTGCTGAAGCCAGTTACCTTCGGAAAAAGAGTTGGTAGCTCTTGATCCGGCAAACAAACCACCGCTGGTAGCGGTGGTTTTTTTGTTTGCAAGCAGCAGATTACGCGCAGAAAAAAAGGATCTCAAGAAGATCCTTTGATCTTTTCTACGGGGTCTGACGCTCAGTGGAACGAAAACTCACGTTAAGGGATTTTGGTCATGAGATTATCAAAAAGGATCTTCACCTAGATCCTTTTAAATTAAAAATGAAGTTTTAAATCAATCTAAAGTATATATGAGTAAACTTGGTCTGACAGttacgccccgccctgccactcatcgcagtactgttgtaattcattaagcattctgccgacatggaagccatcacagacggcatgatgaacctgaatcgccagcggcatcagcaccttgtcgccttgcgtataatatttgcccatggtgaaaacgggggcgaagaagttgtccatattggccacgtttaaatcaaaactggtgaaactcacccagggattggctgagacgaaaaacatattctcaataaaccctttagggaaataggccaggttttcaccgtaacacgccacatcttgcgaatatatgtgtagaaactgccggaaatcgtcgtggtattcactccagagcgatgaaaacgtttcagtttgctcatggaaaacggtgtaacaagggtgaacactatcccatatcaccagctcaccgtctttcattgccatacggaattccggatgagcattcatcaggcgggcaagaatgtgaataaaggccggataaaacttgtgcttatttttctttacggtctttaaaaaggccgtaatatccagctgaacggtctggttataggtacattgagcaactgactgaaatgcctcaaaatgttctttacgatgccattgggatatatcaacggtggtatatccagtgatttttttctccatAACACCCCTTGTATTACTGTTTATGTAAGCAGACAGTTTTATTGTTCATGACCAAAATCCCTTAACGTGAGTTTTCGTTCCACTGAGCGTCAGACCCCGTAGAAAAGATCAAAGGATCTTCCACATTTCCCCGAAAAGTGCCACCTGACGTCTAAGAAACCATTATTATCATGACATTAACCTATAAAAATAGGCGTATCACGAGGCCCTTTCGTCTTCAAGAA

**Note:** (1) DNA sequences in purple are presented as _L_-PHE biosensor: p*mtr*, in which DNA sequences in blue are presented as weak TyrR box (TYR) and DNA sequences in orange are marked as strong TyrR box (PHE); (2) DNA sequences in green are shown as RBS sequence; (3) DNA sequences in red are presented as RFP, encoding for red fluorescent protein.

# Primers used in this study

Table S1. Primers used in this study.

| Primers | Sequence (5’→3’) |
| --- | --- |
| *fruR-N20* | gctcagtcctaggtataatactagtAGCACGAAGCCCAGCTGCCAgttttagagctagaaatagcaagttaaaataaggctagtccg |
| *aroF-N20* | gctcagtcctaggtataatactagtAGATTATCGCCGTCAGCCTGgttttagagctagaaatagcaagttaaaataaggctagtccg |
| *Cm-N20* | gctcagtcctaggtataatactagtTGATGAACCTGAATCGCCAGgttttagagctagaaatagcaagttaaaataaggctagtccg |
| *pheA-N20* | gctcagtcctaggtataatactagtCGGCACTTTGTACGGTTTGCgttttagagctagaaatagcaagttaaaataaggctagtccg |
| *pGRB-REV* | aaaaaagcaccgactcggtgccactttttcaag |
| *pGRB-FOR* | cttcctcgctcactgactcgctg |
| *fruR-out-FF* | caccgagtcggtgcttttttatgttgagcgggaaatcatgctggtg |
| *fruR-out-FR* | tgccgttaataacatagcttgcagtggtc |
| *fruR-out-RF* | aagctatgttattaacggcaaagccaaaacctggtttaacgcgcattaaac |
| *fruR-out-RR* | cgagtcagtgagcgaggaagtaacgttatgaatagctgtaagaaaaaatc |
| *CmR-FOR* | attttacgcaaggggcaatttgatcggcacgtaagaggttcc |
| *CmR-REV* | cgcgtatttttgttcgcggcttacgccccgccctgcc |
| *fruR-R-For-New* | gccgcgaacaaaaatacgcg |
| *fruR-F-Rev-New* | aattgccccttgcgtaaaatgtaaaaac |
| *fruR-MT-FOR* | aagctatgttattaacggcaaagcgaagcaataccgtgtgagcgac |
| *fruR-MT-REV* | gttaaaccaggttttggcttacgcggttcgtccaggcttgccaggac |
| *pheA-out-FF* | caccgagtcggtgcttttttcatccgccaacatgtcgcagac |
| *pheA-out-FR* | ccgttctgccagtaacgctaataatttttcatc |
| *pheA-out-RF* | ttaggggaaatcacccgttc |
| *pheA-out-RR* | cgagtcagtgagcgaggaagtagcctggcaaagcaagttg |
| *pheA-WT-FOR* | tagcgttactggcagaacggcgcgaactggccgtcgaggtgggaaaagc |
| *pheA-WT-REV* | gaacgggtgatttcccctaactctttcaatgctttttgcatttccgctg |
| *aroF-out-FF* | caccgagtcggtgcttttttgcagcctgaaatcatcgcctcttgttgag |
| *aroF-out-FR* | cggagtcattaaaacctgttcgtcggtaatatgtac |
| *aroF-out-RF* | gccttgctgcgtgaaattcatcaggatctgaac |
| *aroF-out-RR* | cgagtcagtgagcgaggaagcaactcaatcgcctcgccgaaacgcttatag |
| *aroF-WT-FOR* | aacaggttttaatgactccggaacaactgaaggccgcttttccattgag |
| *aroF-WT-REV* | tgaatttcacgcagcaaggcatcggtcatttcccagctaatgcaggcatc |
| *vector-FOR* | gtgcatcgcccagtaactgcacacggtgcctgactgcgttagc |
| *vector-REV* | gtttgtcggtgaacgctctcatggaagccggcggcacctcgctaac |
| *RFP-FOR* | agaaagaggagaaatactagatggttagcaaaggcgaagagctgatc |
| *RFP-REV* | gagagcgttcaccgacaaacaacagataaaac |
| *pmtr-FOR* | gcagttactgggcgatgcacagccgcatac |
| *pmtr-REV* | ctagtatttctcctctttctctagtatctagtatgcattgcactgtaccagtacacgagtac |

# References

[1] Webb B, Sali A: Comparative protein structure modeling using MODELLER. Curr Protoc Bioinform. 2016, 54:5.6.1-5.6.37.

[2] Trott O, Olson AJ: Autodock Vina: improving the speed and accuracy of docking with a new scoring function, efficient optimization, and multithreading. J Comput Chem. 2010, 31:455-461.
